# Theory of obstetrics: An epidemiologic framework for justifying medically indicated early delivery

**DOI:** 10.1186/1471-2393-7-4

**Published:** 2007-03-28

**Authors:** K S Joseph

**Affiliations:** 1 Perinatal Epidemiology Research Unit, Departments of Obstetrics & Gynaecology and of Pediatrics, Dalhousie University, Halifax, Nova Scotia, Canada; 2 IWK Health Centre, Halifax, Nova Scotia, Canada

## Abstract

**Background:**

Modern obstetrics is faced with a serious paradox. Obstetric practice is becoming increasingly interventionist based on empirical evidence but without a theoretical basis for such intervention. Whereas obstetric models of perinatal death show that mortality declines exponentially with increasing gestational duration, temporal increases in medically indicated labour induction and cesarean delivery have resulted in rising rates of preterm birth and declining rates of postterm birth. Other problems include a disconnection between patterns of gestational age-specific growth restriction (constant across gestation) and gestational age-specific perinatal mortality (exponential decline with increasing duration) and the paradox of intersecting perinatal mortality curves (low birth weight infants of smokers have lower neonatal mortality rates than the low birth weight infants of non-smokers).

**Discussion:**

The fetuses at risk approach is a causal model that brings coherence to the various perinatal phenomena. Under this formulation, pregnancy complications (such as preeclampsia), labour induction/cesarean delivery, birth, revealed small-for-gestational age and death show coherent patterns of incidence. The fetuses at risk formulation also provides a theoretical justification for medically indicated early delivery, the cornerstone of modern obstetrics. It permits a conceptualization of the number needed to treat (e.g., as low as 2 for emergency cesarean delivery in preventing perinatal death given placental abruption and fetal bradycardia) and a calculation of the marginal number needed to treat (i.e., the number of additional medically indicated labour inductions/cesarean deliveries required to prevent one perinatal death). Data from the United States showed that between 1995–96 and 1999–2000 rates of labour induction/cesarean delivery increased by 45.1 per 1,000 and perinatal mortality decreased by 0.31 per 1,000 total births among singleton pregnancies at > = 28 weeks of gestation. The marginal number needed to treat was 145 (45.1/0.31), showing that 145 excess labour inductions/cesarean deliveries in 1999–2000 (relative to 1995–96) were responsible for preventing 1 perinatal death among singleton pregnancies at > = 28 weeks gestation.

**Summary:**

The fetuses at risk approach, with its focus on incidence measures, provides a coherent view of perinatal phenomena. It also provides a theoretical justification for medically indicated early delivery and reconciles the contemporary divide between obstetric theory and obstetric practice.

## 1. Background

Increases in medically indicated labor induction and cesarean delivery in recent decades have resulted in increases in preterm (< 37 weeks) birth rates, while births at term (37–41 weeks) and postterm (≥ 42 weeks) gestation are also being delivered much earlier than previously [1-10]. However, such changes in obstetric practice are not consistent with obstetric theory since traditional obstetric models of perinatal death show that perinatal mortality rates decrease exponentially as gestational age increases [11-14]. This paper examines the 'paradox of modern obstetrics' [15] and various other conundrums within perinatology and discusses the 'fetuses at risk approach' as a potential solution. The latter approach is an epidemiologic formulation that identifies fetuses as the candidates for perinatal events (as opposed to the traditional obstetric and epidemiologic models that typically focus on newborns as the candidates for perinatal events). The fetuses at risk approach provides a coherent framework for reconciling the diverse set of problems facing perinatology [15,16] and for developing a coherent epidemiologic framework for justifying medically indicated early delivery.

### 1.1. The paradox of modern obstetrics

The cornerstone of modern obstetrics is selective, carefully timed early delivery given fetal compromise (maternal indications sometimes necessitate early delivery as well). Medically indicated labor induction and cesarean delivery are typically employed when the balance of risks and benefits indicate that birth and supportive neonatal care are preferable to an intrauterine environment that is adversely affecting fetal well-being.

**Figure 1:**
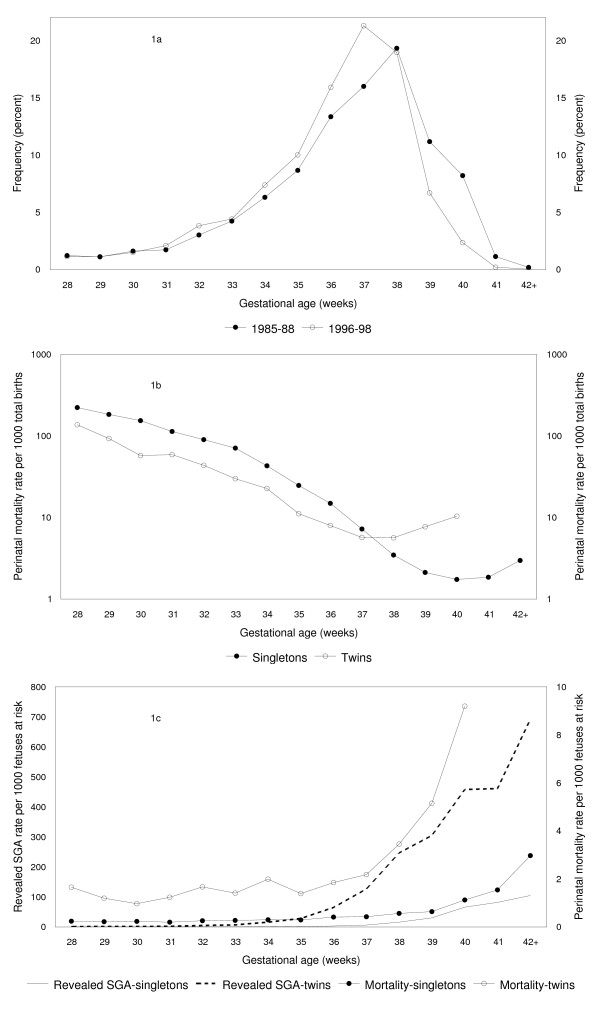
**Gestational age distribution (1a) and gestational age-specific perinatal mortality (1b and 1c) rates, Canada. **Gestational age distribution of twin live births in Canada 1985–88 versus 1996–98 (Figure 1a), conventional calculation of gestational age-specific perinatal mortality rates per 1,000 total births among singletons and twins, Canada 1991–97 (Figure 1b), and gestational age-specific rates of revealed small-for-gestation age (SGA, primary Y-axis) and perinatal death (secondary Y-axis) per 1,000 fetuses at risk among singletons and twins, Canada 1991–97 (Figure 1c). Reprinted with permission [16].

Induction of labor to effect early delivery was introduced in the mid-18th century as a management option for contracted pelvis [17]. In the 1950s, early delivery after 35 weeks gestation was routinely used to prevent stillbirth in severe cases of Rh hemolytic disease [18]. More recently, with advances in the diagnosis of fetal compromise (biophysical profile, umbilical artery Doppler velocimetry, etc) and in neonatal care (antenatal corticosteroids, surfactant, assisted ventilation, etc), rates of medically indicated labor induction and cesarean delivery have increased substantially in industrialized countries at preterm, term and postterm gestation [1-10,19]. The consequent "left-shift" in the population distribution of gestational age at birth (Figure 1a) has been responsible for the well-recognized phenomenon of rising preterm birth rates and declining postterm birth rates in industrialized countries. In Canada, preterm birth rates among twins and higher order multiple births have increased monotonically from approximately 30% in the 1970s, to 40% in the early 1980s, to 50% in the 1990s and to approximately 55% currently [1-4]. Substantial changes have occurred in the gestational age distribution of singletons as well, with increases in preterm birth rates from 5.6 percent in 1981–83 to 6.4 percent in 2000, and declines in postterm birth rates from 6.0 percent in 1981–83 to 1.2 percent in 2000 [1,2,4]. Most of the latter decline in postterm births has occurred due to the introduction of a policy of routine labour induction for postterm pregnancies [10] (although changes in the modality of gestational age ascertainment, from menstrual dating to ultrasound dating, have contributed as well [20]).

However, traditional epidemiologic and obstetric models of perinatal death do not support this iatrogenic increase in early delivery [11-14]. Such models show that the rate of gestational age-specific perinatal mortality (calculated by dividing the number of perinatal deaths at any gestation by the number of total births at that gestation) decreases exponentially as gestational age advances (Figure 1b). Although such models provide a justification for early delivery at ≥ 41 weeks for singletons and at ≥ 39 weeks for twins (Figure 1b), they suggests that a left-shift in the gestational distribution in the preterm or term gestational age range will lead to increases in overall perinatal mortality rates. For instance, early delivery at 34 weeks instead of 36 weeks gestation (or early delivery of singletons at 38 instead of 40 weeks) implies a substantially higher perinatal mortality rate (note log scale, Figure 1b). In fact, the recent left-shift in the gestational age distribution in Canada and in the United States (due to increases in labor induction and cesarean delivery) was accompanied by *a decline *in perinatal mortality [1,4,7-9,21-23].

### 1.2. Other apparently contradictory phenomena in the perinatology

The paradox of modern obstetrics is also evident in relation to cerebral palsy. Although preterm birth is highly associated with cerebral palsy and deemed to be an important cause of cerebral palsy [11,24], the rising rate of preterm birth (especially among twins) has not resulted in an epidemic of cerebral palsy. Related conundrums are evident in the literature on fetal growth restriction [15,16]. The methods used to identify small-for-gestational age (SGA) live births (< 3^rd ^or < 10^th ^percentile of birth weight for gestational age) suggest that a fixed fraction of births (approximately 3% or 10% depending on the cut-off used) are growth restricted at each gestation. Such an implied constancy of the growth restriction rate across gestation is at odds with an exponentially declining rate of gestational age-specific perinatal mortality. Clearly, this incongruence between patterns of in utero growth faltering and death needs to be reconciled, given the known relationship between fetal growth restriction and perinatal death [25,26].

Other problems in the fetal growth literature relate to fetal growth standards. Some fetal growth standards provide unisex reference values [27-30], several are sex-specific [25,31-40] and yet others provide both sex-specific and unisex reference values [41-44]. Of equal concern is the fact that several fetal growth standards are customized for different races [25,31,33-36], parity [31,33,35,40,42], plurality [30,36] and other characteristics [30,33], while others are not [27-29,32,37-39,41,43].

Perhaps the most intriguing of the paradoxes in the perinatal literature is presented by intersecting birth weight- and gestational age-specific perinatal mortality curves. Birth weight-and gestational age-specific perinatal mortality curves intersect [45] when contrasts are made by smoking status, plurality (Figure 1b), race, parity, infant sex, country, etc. This phenomenon was first identified by Yerushalmy [46] who showed that whereas, at low birth weight, infants of smokers have a *lower *neonatal mortality rate than infants of non-smokers, the reverse is true at higher birth weight. Are the low birth weight or preterm infants of smokers more healthy than the low birth weight or preterm infants of nonsmokers? Addressing the paradox of intersecting perinatal mortality curves is important because the resolution of scientific paradoxes often leads to greater substantive insights. The contemporary appeal of traditional models notwithstanding, intersecting perinatal mortality curves (and the other above-mentioned conundrums) suggest that there may be a more compelling perspective on perinatal events.

## 2. Discussion

### 2.1 Problems with traditional models

The conundrums and paradoxes evident in contemporary perinatology are, at least partly, a consequence of the manner in which time related concepts are addressed in traditional models.

#### 2.1.1. Time scales and anchors

Two time scales are commonly used in perinatology and these measure the duration of life in utero (gestational age, which is anchored to the first day of the last menstrual period) and the duration of life after birth (chronologic age, which is anchored to birth). The clinical problems caused by these dual overlapping scales are generally recognized, especially by clinicians in neonatology, who resort to a single scale for expressing age, namely, post-menstrual age or corrected gestational age. Such recognition is also reflected in the evolution of Bronchopulmonary Dysplasia, which was historically defined as a requirement for oxygen at more than 28 days after birth but now refers to a requirement for oxygen or ventilatory support at 36 weeks of post-menstrual age [47]. An important aspect of the use of dual time scales that is not related to duration issues is the qualitative label that is assigned to death depending on whether death occurs before or after the second time scale becomes operational. Thus, a fetus who dies in utero at 38 weeks is a stillbirth but another who dies at 2 weeks of chronologic age after birth at 36 weeks is a neonatal death. Birth has a preeminent position in qualifying life events for reasons that appear to be more sociologic than biologic.

#### 2.1.2. Status of gestational age: determinant versus survival time

Gestational age is often treated as a determinant in perinatal epidemiologic studies. As a determinant, gestational age at birth (and birth weight, which is closely correlated with gestational age) serves as a powerful predictor of death and other adverse perinatal outcomes. However, from an epidemiologic perspective, gestational age is in fact follow up (survival) time and should be treated as such in causal models.

### 2.2. The fetuses at risk approach

**Figure 2:**
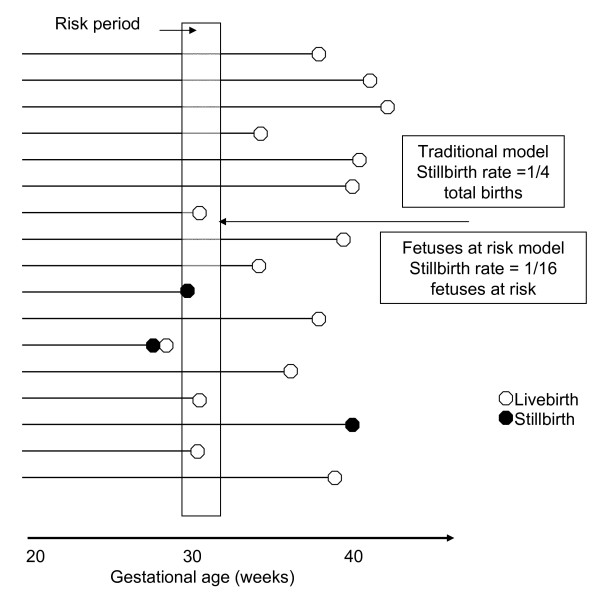
**Schematic depiction of pregnancy course and options for calculating the gestational age-specific stillbirth rate. ***Traditional calculation*: Number of stillbirth at any gestational week/Number of total births at that gestational week = 1/4 = 250 per 1,000 total births. *Fetuses at risk calculation*: Number of stillbirths at any gestational week/Number of fetuses at risk of stillbirth at that gestational week = 1/16 = 63 per 1,000 fetuses at risk.

The problem inherent in calculating traditional gestational age-specific stillbirth rates (e.g., using the number of stillbirths and live births at 32 weeks as the denominator for the stillbirth rate at 32 weeks) and equating these estimates with gestational age-specific stillbirth risk was first identified over 15 years ago [48]. Yudkin et al [48] proposed that all fetuses delivered and undelivered at the gestational age of interest are at risk of fetal death at that gestation and constitute the denominator for calculating the risk of stillbirth at that gestational age (Figure 2). This 'fetuses at risk' formulation for stillbirth is widely recognized and accepted in the literature [49-55], although the traditional formulation has numerous adherents as well [11-14,56]. More recently, Yudkin's formulation [48] has been extended beyond stillbirth to include the estimation of incidence rates for various perinatal phenomena including birth, growth restriction, and perinatal death [15].

#### 2.2.1. Incidence of birth, labour induction, pregnancy complications

**Figure 3:**
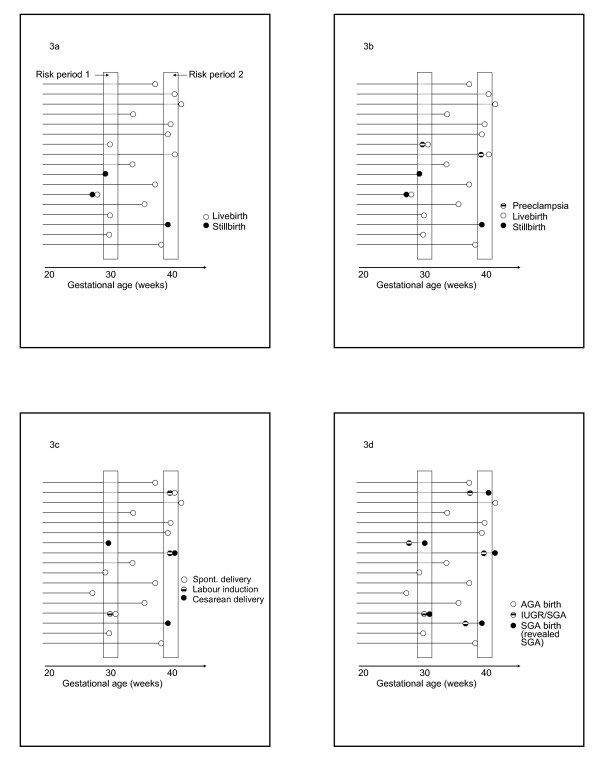
**Schematic depiction of pregnancy course and options for calculating the incidence of various perinatal phenomena. **Schematic depiction of the course of several pregnancies illustrating the options for calculating the gestational age-specific rate (incidence) of stillbirth (Figure 3a), preeclampsia (Figure 3b), obstetric intervention (Figure 3c), and revealed small-for-gestational age (figure 3d). **Figure 3a**: *Traditional calculation*: Number of stillbirths at any gestational week/Number of total births at that gestational week = 1/4 = 250 per 1,000 total births in the first risk period and 1/5 = 200 per 1,000 total births in the second period. *Fetuses at risk calculation*: Number of stillbirths at any gestational week/Number of fetuses at risk of stillbirth at that gestational week = 1/16 = 63 per 1,000 fetuses at risk in the first risk period and 1/6 = 167 per 1,000 fetuses at risk in the second period. **Figure 3b**: *Traditional calculation*: Number of deliveries with preeclampsia at any gestational week/Number of deliveries at that gestational week = 1/4 = 250 per 1,000 deliveries for the first period and 1/5 = 200 per 1,000 deliveries for the second period.*Fetuses at risk calculation*: Number of new cases of preeclampsia at any gestational week/Number of pregnancies at risk of preeclampsia at that gestational week = 1/16 = 63 per 1,000 pregnancies at risk in the first period and 1/6 = 167 per 1,000 fetuses at risk in the second period. **Figure 3c**: *Traditional calculation*: Number of deliveries following labour induction or cesarean delivery at any gestational week/Number of deliveries at that gestational week = 2/4 = 500 per 1,000 deliveries for the first risk period and 3/5 = 600 per 1,000 deliveries for the second period. *Fetuses at risk calculation*: Number of deliveries following labour induction or cesarean delivery at any gestational week/Number of pregnancies at risk of labour induction or cesarean delivery at that gestational week = 2/16 = 125 per 1,000 pregnancies at risk for the first period and 3/6 = 500 per 1,000 pregnancies at risk for the second period.**Figure 3d**: *Traditional calculation*: SGA rate assumed to be uniform 10% or 3% at each gestation depending on cutoff used (10^th ^percentile or 3^rd ^percentile). *Fetuses at risk calculation*: Number of new SGA cases at any gestational week/Number of fetuses at risk of SGA at that gestational week = 1/15 = 67 per 1,000 fetuses at risk for the first risk period and 1/4 = 250 per 1,000 fetuses at risk for the second risk period. *Fetuses at risk calculation for revealed SGA rate*: Number of revealed SGA cases at any gestational week/Number of fetuses at risk of SGA birth at that gestational week = 2/16 = 125 per 1,000 fetuses at risk in the first risk period and 2/6 = 333 per 1,000 fetuses at risk in the second period.

The incidence rate of any pregnancy related event at any gestation is defined as the number of new cases of the event that occur within that gestational week divided by the number of candidates at risk for the event at that gestation. Thus, the incidence of birth (Tables 1, 2) is calculated by dividing the number of births at any gestation by the number of fetuses at risk of birth at that gestation [15,57,58]. The concept is appropriately extended to all relevant perinatal phenomena including the incidence of labor induction, cesarean delivery [15,59] and pregnancy complications (such as hyperemesis gravidarum [60] preeclampsia and chorioamnionitis [61,62], Figure 3). In fact, documenting the incidence pattern of most pregnancy complications over the course of pregnancy has not been undertaken seriously. Although the exact time when a pregnancy complication occurs may sometimes be difficult to ascertain, this is not a sufficient reason for abandoning the study of the incidence patterns of pregnancy complications.

**Table 1:** Numbers and rates of perinatal death, live birth and small for gestational age (SGA) live birth among singletons births, Canada (excluding Ontario), 1991 to 1997.

Gestational age	Stillbirths	Neonatal deaths	Live births	SGA live births	Rates (Conventional)		Fetuses at risk†	Rates (Fetuses at risk)		
							
					SGA (%)	Perinatal mortality per 1,000		Births per 1,000	R-SGA per 1,000	Perinatal mortality per 1,000
34	329	175	10661	1011	9.5	45.9	1583286	6.9	0.6	0.32
35	318	172	18128	1822	10.1	26.6	1572296	11.7	1.2	0.31
36	439	249	41962	4226	10.1	16.2	1553850	27.3	2.7	0.44
37	451	269	87566	9104	10.4	8.2	1511449	58.2	6.1	0.48
38	552	364	232039	22163	9.6	3.9	1423432	163.4	15.6	0.64
39	522	359	356922	35792	10.1	2.5	1190841	300.2	30.2	0.74
40	601	464	536302	54709	10.2	2.0	833397	644.2	65.9	1.28
41	304	219	245665	24104	9.8	2.1	296494	829.6	81.7	1.76
≥ 42	92	77	50433	5250	10.5	3.3	50525	1000.0	104.8	3.34

Total‡	8694	5562	1614531	160429	10.0	8.8	1623225	1000.0	100.0	8.70

Note: Perinatal mortality includes stillbirths and neonatal deaths. Live births at each gestation served as the denominator for the conventional SGA rate and total births at each gestational week served as the denominator for conventional perinatal mortality rate. Under the fetuses at risk approach, birth, revealed SGA (R-SGA) and perinatal mortality rates were calculated using the number of fetuses at risk at that gestation as the denominator for the rate.

† Calculated by summing the number of fetuses who delivered at that and subsequent gestational weeks.

‡ All gestational ages, including those < 34 weeks and those with missing gestational age.

**Table 2:** Numbers and rates of perinatal death, live birth and small for gestational age (SGA) live birth among twin births, Canada (excluding Ontario), 1991 to 1997.

Gestational age	Stillbirths	Neonatal deaths	Live births	SGA live births	Rates (Conventional)		Fetuses at risk†	Rates (Fetuses at risk)		
							
					SGA (%)	Perinatal mortality per 1,000		Births per 1,000	R-SGA per 1,000	Perinatal mortality per 1,000
34	42	23	2513	447	17.9	25.4	29170	87.6	15.5	1.99
35	28	14	3302	740	22.5	12.6	26615	125.1	28.0	1.39
36	28	23	5372	1491	27.8	9.4	23285	231.9	64.5	1.85
37	29	15	6835	2249	33.0	6.4	17885	383.8	126.7	2.18
38	30	11	6720	2695	40.2	6.1	11021	612.5	246.5	3.45
39	15	7	2843	1291	45.5	7.7	4271	669.2	304.8	5.15
40	11	2	1246	641	51.5	10.3	1413	889.6	458.2	9.20
41	1	0	129	71	55.5	7.7	156	833.3	461.0	6.41
≥ 42	0	0	26	18	69.2	0.0	26	1000.0	692.3	0.00

Total‡	703	869	34944	10325	29.9	44.1	35647	1000.0	298.8	44.1

Note: Perinatal mortality includes stillbirths and neonatal deaths. Live births at each gestation served as the denominator for the conventional SGA rate and total births at each gestational week served as the denominator for conventional perinatal mortality rate. Under the fetuses at risk approach, birth, revealed SGA (R-SGA) and perinatal mortality rates were calculated using the number of fetuses at risk at that gestation as the denominator for the rate.

† Calculated by summing the number of fetuses who delivered at that and subsequent gestational weeks.

‡ All gestational ages, including those < 34 weeks and those with missing gestational age.

#### 2.2.2. The incidence of growth restriction

The incidence of growth restriction is a good example of an index whose estimation presents a challenge. Although routine obstetric practice includes screening for and diagnosis of growth restriction, the technology is insufficiently advanced to permit valid and complete ascertainment of all new cases at each gestation [63-65]. Given this limitation, an alternative index, namely, the incidence of revealed SGA (Tables 1, 2, Figure 1c, Figure 3) may be calculated by dividing the number of SGA births at any gestation by the number of fetuses at risk of SGA birth at that gestation [15,16,57-59]. The primary utility in estimating this proxy incidence rate is in order to approximate the incidence *pattern *of growth restriction with increasing gestational duration i.e., to estimate whether it increases, decreases or remains constant. The measure cannot provide the absolute rate of SGA at any gestation since the numerator of the index is dependant on birth (hence the term "revealed"). Improvements in technology will permit quantification of the absolute rate (incidence) of growth restriction at each gestation in the future.

#### 2.2.3. The incidence of perinatal death

**Figure 4:**
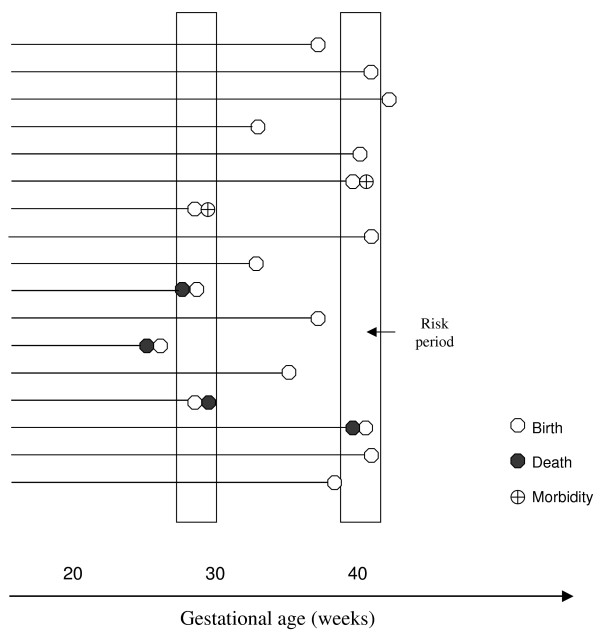
**Schematic depiction of the survival analysis (obstetric) model for perinatal death or serious neonatal morbidity. ** Schematic depiction of survival analysis model for perinatal death or serious neonatal morbidity with censoring at death or birth (whichever occurs earlier). Perinatal death and serious neonatal morbidity are assigned to the point of birth. In the first risk period, there are 16 fetuses at risk of perinatal death or serious neonatal morbidity, 3 births, 1 stillbirth, 1 neonatal death and 1 case of serious neonatal morbidity. In the second risk period, there are 7 fetuses at risk, 6 births, 1 stillbirth and 1 case of severe neonatal morbidity. Under the conventional calculation, with perinatal mortality defined as the number of perinatal deaths within any period divided by the number of total births in that period, the perinatal mortality/morbidity rate is 3/3 in the first risk period and 2/6 in the second. Note increase in denominator and decrease in rate from the first risk period to the second risk period (from 100% to 33%). Under the fetuses at risk formulation, with perinatal mortality defined as the number of perinatal deaths in any period divided by the number of fetuses at risk of perinatal death in that period, the perinatal mortality/morbidity rate is 3/16 in the first risk period and 2/7 in the second risk period. Note decrease in denominator and increase in rate from the first to the second risk period (from 19% to 29%).

The fetuses at risk approach for stillbirth is a survival analysis model with censoring of subjects (fetuses) at birth. This gestational age-specific stillbirth calculation provides estimates of the cumulative incidence of fetal death at each week of gestation and approximates the incidence density (hazard) of stillbirth. The extended fetuses at risk model integrates perinatal death and serious neonatal morbidity (e.g., severe respiratory distress syndrome, severe intraventricular hemorrhage, etc) into a single framework (Figure 4) since these events all have their origins in pregnancy, labour or birth [15,16,66,67]. This is consistent with principles ingrained in routine obstetric practice and state-of-the-art clinical trials [68,69] where the definitive obstetric outcome embraces perinatal mortality and serious neonatal morbidity. Similarly, with recent literature [70-72] suggesting that cerebral palsy has a predominantly prenatal origin (i.e., critical neurologic injury occurs before birth), this outcome is also assigned to the point of birth, despite being diagnosed years later. Combining stillbirth, neonatal death and serious neonatal morbidity into a single composite outcome is consistent with traditions in obstetrics and is justified by the broadly overlapping multifactorial etiology that characterizes these distinct entities [15].

As mentioned, the extended fetuses at risk model most deviates from traditional models with respect to events that occur after birth and yet have a prenatal etiology. Under the traditional model of perinatal death, neonatal deaths occur among infants in the first month after birth and the unborn fetus is not a candidate for neonatal death. However from a broad biological, obstetric and ultimately epidemiologic point of view, a fetus at any gestation is at risk of stillbirth and neonatal death at that gestation. If one considers a woman at 28 weeks gestation with severe preeclampsia and fetal compromise, the risk of stillbirth is easy to conceptualize. The risk of neonatal death is substantial as well and can follow either premature labour or medically indicated delivery. The same risks apply in concept to a woman with a healthy pregnancy at 28 weeks gestation, despite the magnitude of the risks being considerably smaller [15]. Thus, although neonatal deaths literally occur among infants, fetuses can be considered candidates for neonatal death as well. This is analogous to the calculation of age-specific rates of death from breast cancer. Such rates are calculated using all women in the population as the denominator (i.e., as candidates for death from breast cancer), although one could argue that death from breast cancer can only occur among women with breast cancer.

The extended fetuses at risk formulation of death provides 2 alternative models that treat time per epidemiologic principles (as survival time and on a single time scale). In the first model, namely, the comprehensive model of death, time is measured on the scale of post-menstrual (or post conceptional) age with fetuses/infants censored at death [15]. Birth is ignored as a event (for truncating the original time scale) and no distinction is made between deaths that occur before and after birth. The epidemiologic risk set at any point in post-menstrual time is constituted by the fetuses/infants at risk of death at that point in time [15]. When this framework is integrated into a proportional hazards model, birth may be introduced as a time-dependant covariate with time-varying effects [73]. In the second model, namely, the obstetric model of death, time is measured on the scale of gestational age with fetuses censored at birth or death. All deaths that have their origins in prenatal or labour and delivery events are deemed relevant to obstetrics. Thus, as per traditions in obstetrics, stillbirths and neonatal deaths (and serious neonatal morbidity) are assigned to the point of birth. The epidemiologic risk set for such obstetric outcomes is constituted by the fetuses at risk for such events, namely, all unborn fetuses at the gestational age in question (Figure 4).

#### 2.2.4. Reconciling diverse perinatal conundrums

The fetuses at risk formulation brings coherence to the study of perinatal phenomena. It shows that the incidence of pregnancy complications such as preeclampsia and chorioamnionitis increases as gestational age advances [61,62]. Revealed SGA rates also increase with increasing gestational age (Figure 1c) and presage the rise in perinatal mortality rates [15,16,57-59]. Gestational age-specific perinatal mortality curves do not intersect in comparisons by smoking status, plurality (Figure 1c), race, parity, infant sex, etc [57-59]. Smokers have higher rates of revealed SGA and perinatal death than non-smokers at all gestational ages [58] and twins [57] have higher rates of revealed SGA and perinatal death than singletons at all gestational ages (Figure 1c). Similarly, the incidence of birth, labor induction and cesarean delivery show patterns that are congruent with patterns of revealed SGA and death [15,57-59,67]. The rising patterns of gestational age-specific revealed SGA and perinatal death also offer a preliminary justification for medically-indicated early delivery. Finally, the fetuses at risk approach provides insights into issues as diverse as the etiology of cerebral palsy [74] and the need for customized fetal growth standards [59,75]. Specifically, it shows that the rate of critical neurologic injury that causes cerebral palsy increases with advancing gestational age [74] and suggests that the pregnancy complications (which precede preterm birth) are the cause of cerebral palsy (and not preterm birth itself). With regards to fetal growth standards, the fetuses at risk formulation shows that perinatal mortality patterns are consistent with separate fetal growth standards for males and females but not with the available separate standards for blacks and whites in the United States [59].

The fetuses at risk formulation faces its most serious challenge from the traditional idea that perinatal mortality declines as gestational age increases. Indeed, this latter inference appears intuitive and is corroborated by the readily apparent relationship between birth weight and perinatal mortality. Despite the socially important prognostic purpose served by the traditional model of perinatal death, it is not appropriate as a causal model. The use of dual overlapping time scales for life in utero (gestational age) and after birth (chronologic age) and the truncation of the full biologic continuum (as in the calculation of neonatal mortality rates using live births at a particular gestational age as the denominator) is problematic on the level of first principles and also because it is responsible for numerous paradoxes and conundrums [15]. The entire mortality experience of a cohort of fetuses (as documented on single time scale) is of interest, irrespective of whether death precedes or follows birth (see Figure 4).

As for explaining the rise in growth restriction and perinatal mortality rates with increasing gestation, one can speculate that the ability of the utero-placental system to support the fetus declines with increasing gestational age. Rising rates of growth restriction (as reflected in rising rates of revealed SGA) and perinatal death with increasing gestational duration reflect increases in the incidence of pregnancy complications such as preeclampsia and chorioamnionitis [61,62] and also other stochastic processes that adversely affect vascular function within the utero-placental system.

The above-mentioned arguments suggest that the traditional and fetuses at risk models serve vastly different purposes. The distinction between descriptive versus causal models is particularly relevant in this context [76]; traditional models which truncate the biologic continuum are better viewed as descriptive (noncausal) models which are ideal for setting prognosis at birth, while the fetuses at risk formulation represents a causal model that yields biologic insights and provides the basis for obstetric intervention [77].

### 2.3. An epidemiologic framework for medically indicated early delivery

Developing an explicit epidemiologic framework for justifying medically indicated early delivery is important in order to avoid conflicts between obstetric theory and practice [16]. Thus, in the absence of appropriate obstetric theory, population increases in preterm birth (occurring secondary to increases in medically indicated preterm birth) may be viewed as adverse developments under the traditional theoretical framework. This would lead to a discounting of the perinatal mortality reductions that are a consequence of recent changes in the management of compromised fetuses at preterm gestation [1,4,7-9,21-23]. Also, the obstetric literature needs to be more articulate with respect to the number of labor inductions and cesarean deliveries that are needed to prevent one perinatal death or serious neonatal morbidity (given a particular domain/indication). The proposed epidemiologic framework based on the fetuses at risk model is illustrated below using live births and stillbirths in the United States between 1995–1996 and 1999–2000 (National Center for Health Statistics perinatal mortality data file for all states and the District of Columbia). Perinatal mortality was defined to include stillbirths and neonatal deaths [11] but excluded perinatal deaths due to congenital anomalies (in order to eliminate the potential effect of temporal increases in prenatal diagnosis and pregnancy termination for major congenital anomalies [78]).

#### 2.3.1. Incidence of medically indicated early delivery, birth, revealed SGA, and death

**Figure 5:**
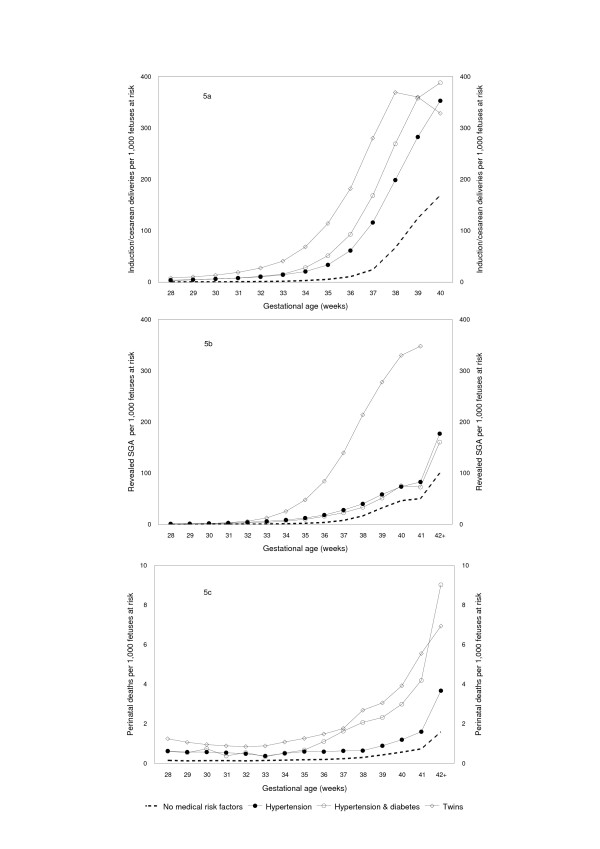
**Incidence of labor induction/cesarean delivery, revealed small-for-gestation age (SGA) and perinatal death, United States 1999–2000.** Incidence of labor induction and/or cesarean delivery (Figure 5a), incidence of revealed SGA (Figure 5b) and incidence of perinatal death (Figure 5c) at 28 weeks gestation and over, among pregnancies with no medical risk factors, hypertension, hypertension and diabetes and twins, United States 1999–2000. Hypertension includes chronic and pregnancy-associated hypertension and eclampsia (National Center for Health Statistics definitions).

The rate of labor induction and/or cesarean delivery increased with increasing gestational age (Figure 5a), being lowest among pregnancies with no medical risk factors and higher among pregnancies with complications. The incidence of birth showed a similar pattern. The incidence of revealed SGA rose with increasing gestational age and was highest among twins and lowest among uncomplicated pregnancies (Figure 5b). Rates of perinatal death also increased with increasing gestational age and patterns were generally consistent with clinical expectation and patterns of revealed SGA (Figure 5c).

#### 2.3.2. Number needed to treat

The number needed to treat (NNT), an index widely used in therapeutics as part of risk-benefit equations, is insufficiently articulated in connection with medically indicated early delivery [79]. This is in part because conventional models of perinatal mortality imply that early delivery is associated with an increased rate of perinatal death (Figure 1b). Nevertheless, the concept of the NNT remains as relevant and critical in the context of medically indicated early delivery as elsewhere in medicine. The NNT for medically indicated early delivery (given a specific indication) may be defined as the reciprocal of the difference between the rate of perinatal mortality or serious neonatal morbidity given no obstetric intervention and the rate of perinatal mortality or serious neonatal morbidity given medically indicated early delivery.

#### 2.3.3. Number needed to treat under different scenarios

The clinical scenarios described below are characterized by varying background rates of perinatal mortality (which substantially modify the NNT). The first scenario involves an obstetric emergency (e.g., placental abruption with fetal bradycardia) where a rapid absolute increase in the (incidence density) rate of perinatal death is anticipated over a short time span (minutes). It is expected that an emergency cesarean delivery carried out within 15–30 minutes will prevent perinatal death in more than half the fetuses [80]. This implies an NNT of approximately 2 or less. Similarly, in a second scenario involving a serious pregnancy complication and fetal compromise (e.g., severe preeclampsia with fetal growth restriction), an expected perinatal mortality reduction due to labor induction and/or cesarean delivery (as opposed to no intervention) of about 100 to 200 per 1,000 fetuses implies an NNT of 5 to 10. A third scenario involves routine delivery of twin pregnancy at 38 weeks gestation. If routine delivery at 38 weeks (relative to no obstetric intervention) reduces the rate of perinatal mortality by about 5 per 1,000 fetuses, this implies an NNT of 200. The final scenario involves routine delivery at 41 weeks gestation given an uncomplicated singleton pregnancy. If the difference between perinatal mortality given routine delivery at 41 weeks versus spontaneous delivery without obstetric intervention is approximately 1 per 1,000 fetuses at risk, this implies an NNT of 1,000. In other words, 1,000 routine early deliveries at 41 weeks gestation (through labor induction and/or cesarean delivery) would prevent one perinatal death.

#### 2.3.4. Problems with the NNT calculation

Virtually all estimates used in the NNT calculations above are speculative even if they represent more or less reasonable approximations. In fact, most inputs into the NNT calculation cannot be estimated given current standards of care since the decision not to intervene is such situations (e.g., severe preeclampsia with fetal compromise) would constitute a breach of ethical standards.

#### 2.3.5. Marginal NNT calculation

The marginal NNT, which measures the effect of increases in medically indicated early delivery (beyond standard rates of medically indicated early delivery), is an alternative measure that is directly pertinent to obstetric practice. In this calculation, a temporal increase in medically indicated early delivery is set against the change in perinatal mortality in any particular domain.

Table 3 shows temporal changes in the incidence of obstetric intervention and perinatal death in the United States between 1995–96 and 1999–2000. The rate of labor induction and/or cesarean delivery among singleton pregnancies ≥ 28 weeks of gestation increased by 45.1 per 1,000 fetuses, from 339.4 per 1,000 fetuses in 1995–96 to 384.5 per 1,000 fetuses in 1999–2000 (P < 0.0001, Table 3). During the same period, the rate of perinatal death (excluding deaths due to congenital anomalies) decreased by 0.31 per 1,000 fetuses from 3.95 to 3.64 per 1,000 fetuses at ≥ 28 weeks of gestation (P < 0.0001). This yielded a marginal NNT rate of (45.1/0.31) or 145. Thus, 145 additional labor inductions/cesarean deliveries in 1999–2000 (relative to 1995–96) were responsible for preventing 1 perinatal death among singletons ≥ 28 weeks gestation. Marginal NNT estimates for specific subpopulations differed from those obtained for all singletons, being as low as 32 among twins ≥ 28 weeks and as high 927 among singletons ≥ 34 weeks with hypertension (Table 3).

**Table 3:** Rates of labor induction and/or cesarean delivery, perinatal mortality (excluding congenital malformations) and the marginal number needed to treat in order to prevent one perinatal death in various subpopulations, United States, 1995–96 and 1999–2000.

Population	Labor induction/cesarean deliveries per 1,000 fetuses			Perinatal deaths*/1,000 fetuses			Number needed to treat (marginal)
	1995–96	1999–00	Change†	1995–96	1999–00	Change	
Singletons ≥ 28 weeks, all	339.4	384.5	45.1	3.95	3.64	0.31‡	145
≥ 28 weeks, no medical risk factors	294.6	337.7	43.0	2.98	2.68	0.29‡	146
≥ 28 weeks, with hypertension	662.4	697.6	35.2	6.96	6.76	0.20	180
≥ 28 weeks, with diabetes	532.4	578.4	46.0	6.67	6.49	0.18	257
≥ 28 weeks, with hyp. and diabetes	744.6	779.3	34.7	8.83	9.96	-1.13	-31
Twins ≥ 28 weeks, all	636.5	685.3	48.8	12.13	10.63	1.50‡	32
Singletons ≥ 34 weeks, all	338.3	383.2	44.8	2.66	2.42	0.24‡	188
≥ 34 weeks, no medical risk factors	294.5	337.5	42.0	2.09	1.86	0.23‡	181
≥ 34 weeks, with hypertension	654.1	689.5	35.1	3.91	3.87	0.04	927
≥ 34 weeks, with diabetes	533.3	579.8	45.7	5.25	5.14	0.11	403
≥ 34 weeks, with hyp. and diabetes	740.9	776.6	35.0	6.86	7.29	-0.43	-81
Twins ≥ 34 weeks, all	634.8	685.9	48.7	6.68	6.16	0.53	92

* excluding perinatal deaths due to congenital malformations

† All temporal changes in labor induction/cesarean delivery rates were statistically significant P < 0.0001.

‡ Temporal changes in perinatal death rates statistically significant P < 0.0001.

Hypertension includes chronic and pregnancy-associated hypertension and eclampsia (National Center for Health Statistics definitions).

Such marginal NNT calculations are analogous to calculations based on randomized trials which contrast routine induction of labor vs selective induction of labor at or beyond term [81] or those which contrast aggressive vs expectant management given severe preeclampsia before term gestation [82]. A meta-analysis [81] of studies on the former issue showed that routine induction of labor reduced perinatal death rates several-fold (odds ratio of 0.20, 95% confidence interval 0.06 to 0.70). This implies an NNT of 1,250 for routine induction of labour at or beyond term gestation, assuming a perinatal mortality rate of 1.0 per 1,000 fetuses at risk following selective labor induction.

#### 2.3.6. Limitations

The proposed framework is based on two important assumptions. First, medically indicated early delivery is considered the final pathway for obstetric intervention. Thus, increases in labor induction and cesarean delivery are credited with preventing perinatal death even though such early delivery was facilitated by improved methods for assessing fetal well-being and supportive neonatal care. Early delivery is thus viewed as a therapeutic package which subsumes antenatal monitoring, diagnosis of fetal well-being, supportive neonatal care and other interventions that permit higher rates of early delivery to rescue compromised fetuses from a hostile intrauterine environment.

A second assumption is that temporal increases in labor induction and cesarean delivery rates and declines in perinatal mortality rates reflect true changes in obstetric practice (rather than changes in population characteristics). It is possible that changes in maternal characteristics (such as increases in older maternal age and pre-pregnancy obesity [83,84]) may have been partly responsible for changes in labor induction, cesarean delivery and perinatal mortality rates in the United States between 1995–96 and 1999–2000. Although such changes are unlikely to have affected the results substantially (since the study interval was only 4 years), regression adjustment can be used to address this issue where necessary.

Current limitations of the fetuses at risk approach include an inability to precisely document the incidence of fetal growth restriction. This is because diagnosis of growth faltering in utero, although much facilitated in recent decades through ultrasonographic means, remains inaccurate and essentially unavailable at the population level [63-65]. The alternative index of revealed SGA [15] is useful but limited by its relationship to birth rate patterns. Further developments in ultrasound technology are needed so that incidence rates can be estimated more accurately based on an identification of all new cases of growth restriction (in utero). Another approximation in the fetuses at risk approach relates to the timing of the pathologic process or event. Assigning events such as neonatal death and serious neonatal morbidity to the moment of birth often involves a systematic overestimation of the timing of the critical pathologic process or event [74]. The systematic nature of the problem means that the incidence patterns of perinatal mortality and morbidity are not seriously affected, however.

## 3. Summary

The cornerstone of modern obstetrics, namely, early delivery given fetal compromise, cannot be reconciled with traditional models of perinatal mortality which show that perinatal death rates decline exponentially as gestational duration increases. On the other hand, the fetuses at risk approach, which shows that pregnancy complications, revealed SGA and perinatal death rates increase with increasing gestational age, provides a justification for medically indicated early delivery and also resolves several prevailing conundrums in the perinatal field. Although inputs for estimating therapeutic indices related to medically indicated early delivery (such as the NNT) cannot be obtained for ethical reasons, it is possible to retrospectively estimate the marginal NNT associated with medically indicated early delivery. This provides an estimate of the number of additional medically indicated early deliveries that were required to prevent one perinatal death. On a more general level, the traditional model of perinatal death and the fetuses at risk approach are best viewed as serving different purposes; the former is suited for setting prognosis at birth while the latter provides a causal framework and the basis for obstetric intervention.

## Competing interests

The author(s) declare that they have no competing interests.

## Authors' contributions

KSJ proposed the thesis, carried out the analysis and wrote the manuscript. The paper was presented at a plenary session of the 2004 Annual Meeting of the Society for Pediatric and Perinatal Epidemiology, Salt Lake City, Utah.

## Pre-publication history

The pre-publication history for this paper can be accessed here:


